# Herpes Simply Strikes Back: A Case of Recurrent Esophagitis in an Immunocompetent Patient

**DOI:** 10.7759/cureus.107118

**Published:** 2026-04-15

**Authors:** Mohammed Y. Youssef, Andrea Brestel, Hafsa Khan, Asim Usman, Jaison John

**Affiliations:** 1 Internal Medicine, Arkansas Colleges of Health Education, Greenville, USA

**Keywords:** antiviral therapy, herpes simplex virus esophagitis, hsv esophagitis, immunocompetent patients, odynophagia, recurrence

## Abstract

Herpes simplex virus esophagitis (HE) is a commonly observed disease in immunocompromised individuals, but is rare in immunocompetent individuals and often misdiagnosed due to overlapping features with other causes of esophagitis. Delay in diagnosis and appropriate treatment can lead to severe complications such as esophageal ulceration, resulting in bleeding, perforation, fistulas, food impaction, and strictures. We report a rare case of recurrent HE in an immunocompetent male initially misdiagnosed as *Candida *esophagitis. This case underscores the importance of considering HSV in the differential diagnosis of esophageal ulcerations, highlights the potential for recurrence in immunocompetent hosts, and emphasizes the need for accurate diagnosis, timely antiviral therapy, and patient education to prevent complications.

## Introduction

Herpes simplex virus esophagitis (HE) is a clinically significant inflammatory disease of the esophagus most often caused primarily by herpes simplex virus type 1 (HSV-1) infection, with less frequent involvement of HSV-2. HSV represents approximately 10% of infectious esophagitis, while the majority of infectious esophagitis cases are attributed to *Candida* (88%) [1]. It is most often seen in the immunocompromised population and rarely seen in immunocompetent individuals [2]. HE presents with nonspecific symptoms such as dysphagia, chest/epigastric pain, and odynophagia, which may contribute to underdiagnosis given the overlap of similar symptoms with other etiologies like gastroesophageal reflux disease (GERD) or *Candida *infection. Additionally, other underlying esophageal diseases can sometimes occur simultaneously with HE infections, which further confounds the ability to properly diagnose HE [2,3]. Typically, HE occurrence in immunocompetent individuals is either a self-limiting primary infection with an acute onset [4] or resultant from transient immunosuppression from steroid use [5,6]. While milder cases of primary HSV infection can resolve with supportive management, the use of antivirals (acyclovir, famciclovir, and valacyclovir) is indicated in patients with severe odynophagia and tissue damage, as it hastens healing by preventing the synthesis of viral DNA to reduce HSV replication [7,8]. Delayed diagnosis and delayed treatment can lead to the development of esophageal ulceration and subsequent severe bleeding, esophageal perforation, tracheoesophageal fistula, food impaction, and strictures [9]. Herein, we present the rare case of recurrent HSV esophagitis in an immunocompetent male without a recent history of steroid use.

This article was previously presented as a meeting abstract at the American College of Gastroenterology (ACG) 2025 Annual Scientific Meeting on October 26-29, 2025, in Phoenix, Arizona [10].

## Case presentation

**Figure 1:**
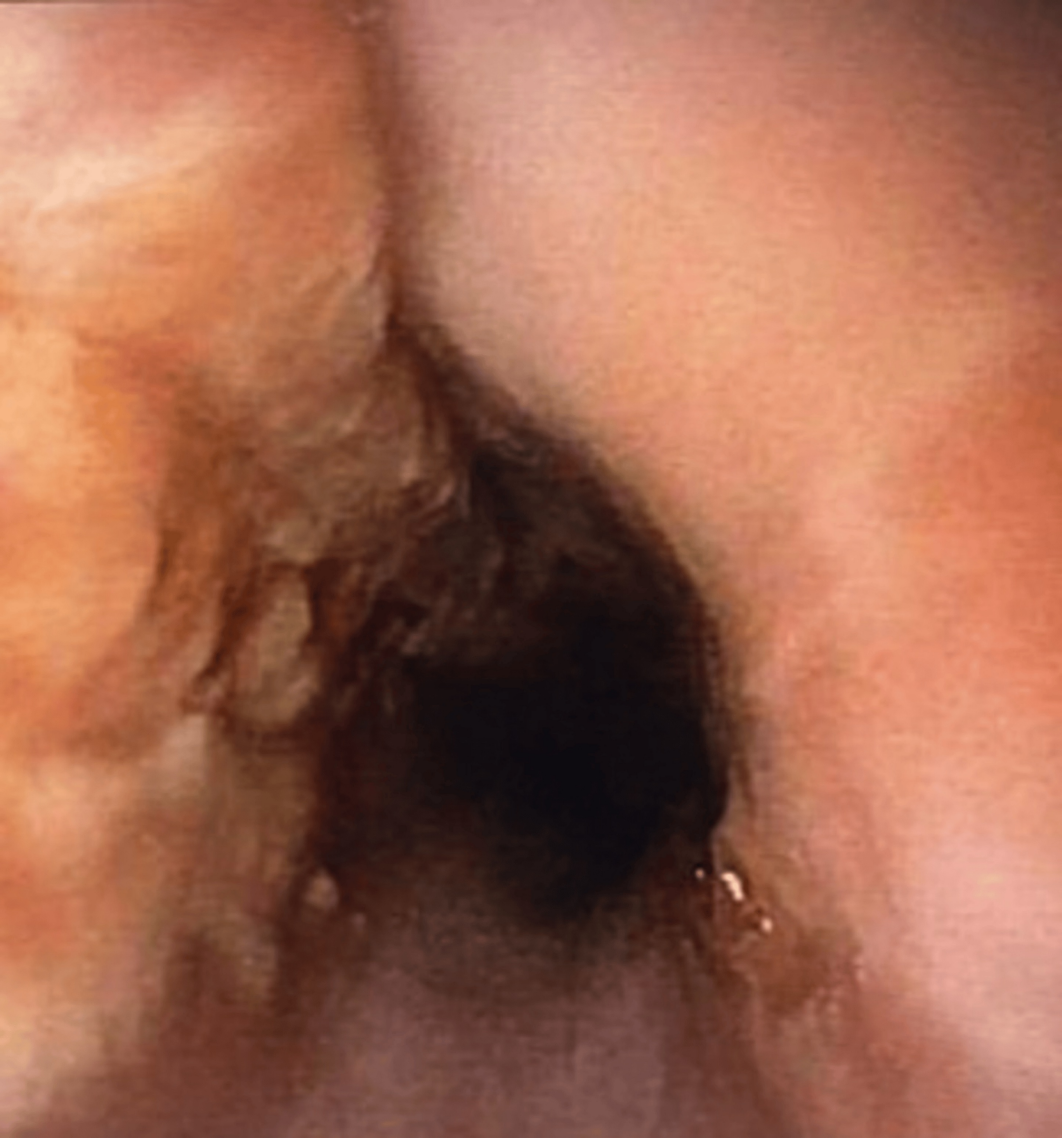
White patchy plaques observed in the middle third of the esophagus

**Figure 2:**
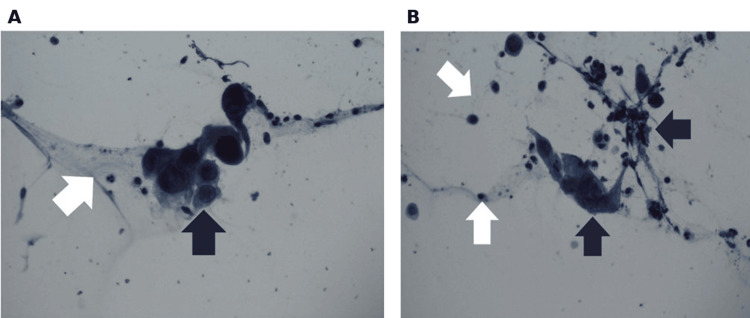
A Papanicolaou (Pap) test with blue staining of an esophageal biopsy sample shows basal cell hyperplasia and inflammatory changes with increased blue stain uptake (black arrow) compared to normal tissue (white arrow) (1 mm field diameter).

A gentleman in his early 30s with a significant history of hypertension, bipolar disorder, and seizures, and with no known history of immunodeficiency, initially presented to the ED with a nine-day history of hematemesis, severe epigastric pain, and melena. He was clinically diagnosed with esophagitis and admitted to the hospital. He was initiated on pantoprazole and underwent an esophagogastroduodenoscopy (EGD). Gross examination of the esophagus revealed white patchy plaques in the middle third and lower third of the esophagus, which suggested *Candida *esophagitis versus the "volcano-like" appearance typically seen with HE ulcers (Figure 1). The patient was then started on fluconazole; however, the sampled biopsies resulted in a negative for *Candida *with potassium hydroxide (KOH) prep testing. Three days later, the finalized biopsy report confirmed a diagnosis of HE instead, with a Papanicolaou (Pap)-stained smear that resulted in a positive diagnosis for HSV with evidence of scattered epithelial cells containing nuclear inclusions characterized by homogenization of the nuclear chromatin, multinucleation, and molding of the nuclei in the background of mature squamous cells and acute inflammatory cells (Figures 2A, 2B). The HIV workup with HIV 1 and HIV-2 antibody differentiation immunoassay and HIV P24 antigen test was negative, the WBC was within normal limits (reference range: 4,500-11,000 cells/µL), and the patient had no known history of autoimmune disease. Fluconazole was discontinued, and the patient was started on oral acyclovir 400 mg thrice a day (TID) for 10 days for treatment of an initial episode of esophageal HSV without concurrent HIV infection. After a total of five days inpatient, the patient was stabilized and discharged home with instructions to complete the acyclovir.

**Figure 3:**
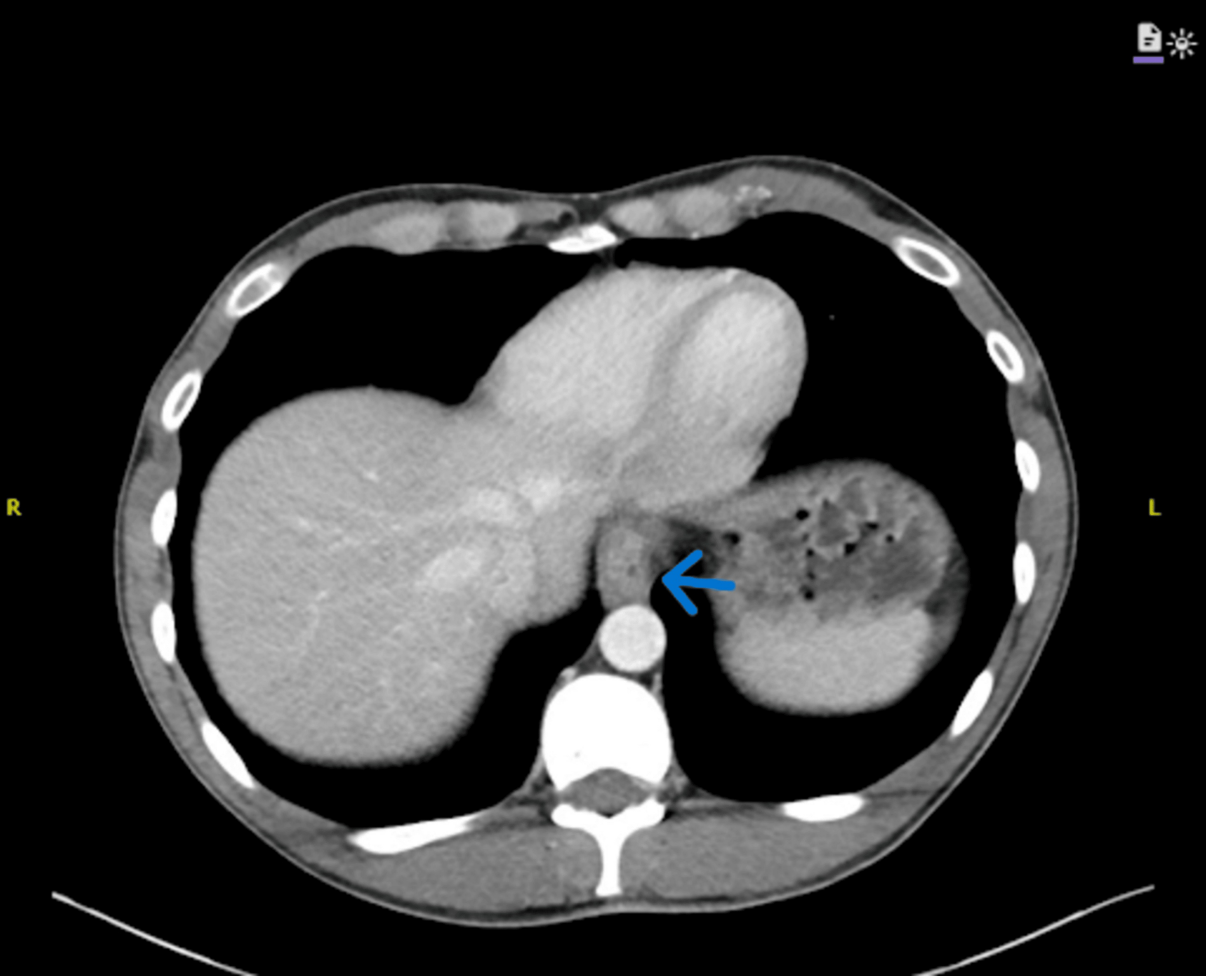
Non-contrast CT demonstrates thickening of the distal esophagus (arrow).

**Figure 4:**
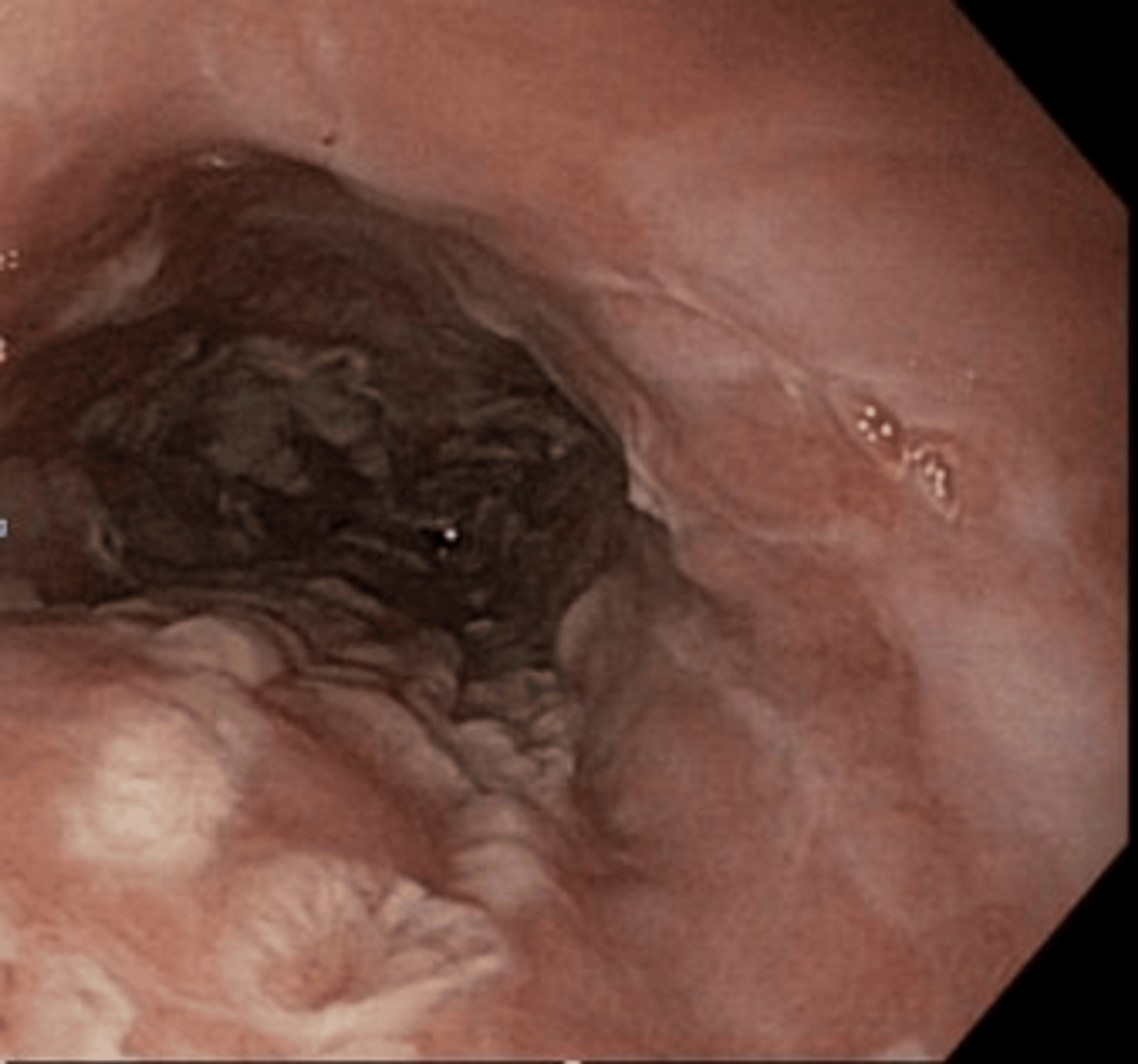
The endoscopic image shows severe herpetiform esophagitis characterized by ulcerated, necrotic-appearing mucosa and friability in the upper third of the esophagus.

**Figure 5:**
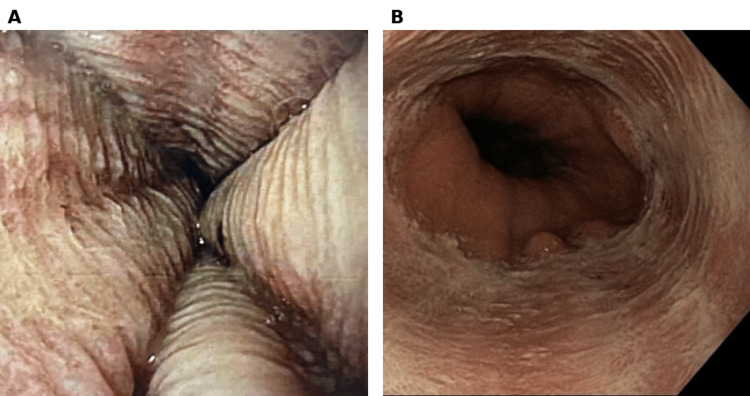
Endoscopic images showing a circular pattern of severe diffuse herpes simplex virus esophagitis involving the middle third (A) and distal third (B) of the esophagus

**Figure 6:**
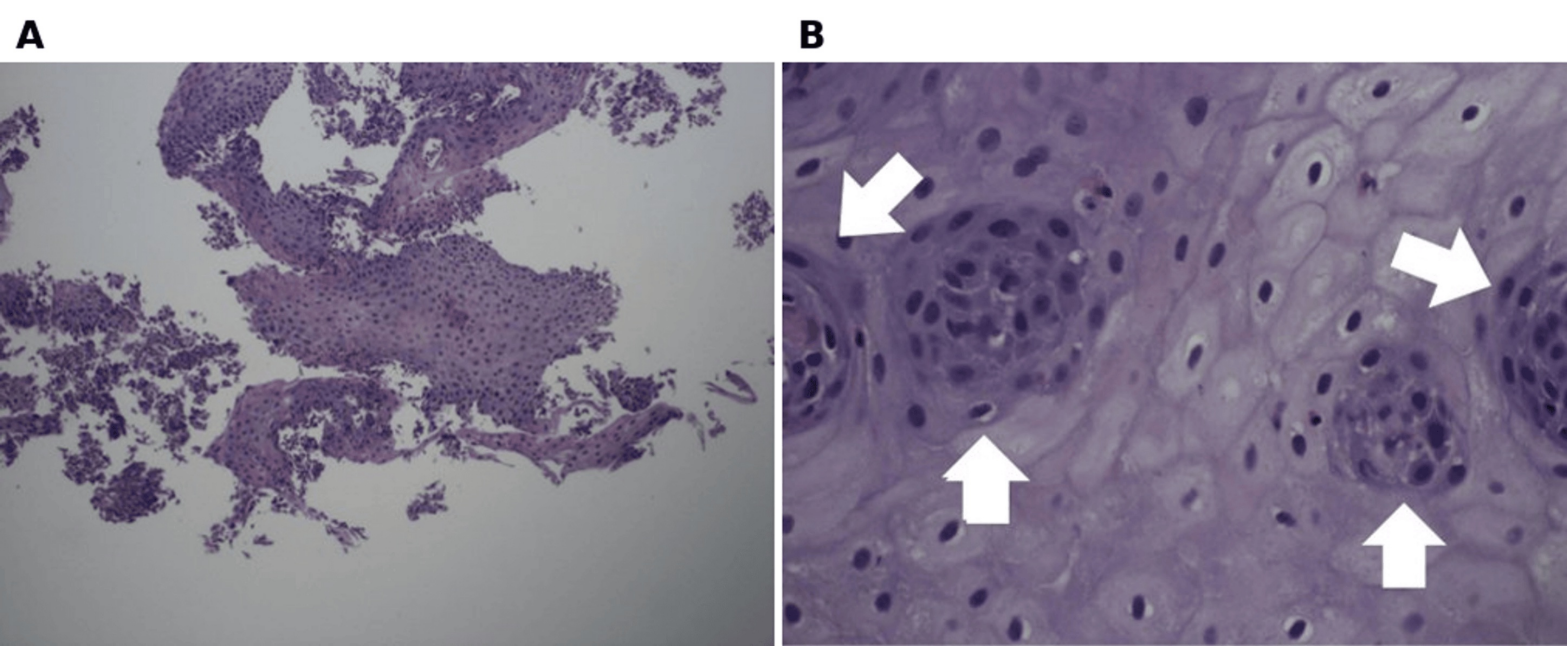
Low-power hematoxylin and eosin (H&E) staining showing basal cell hyperplasia and inflammatory changes (1 mm field diameter) (A). High-power H&E staining showing multinucleated giant cells (white arrows) with surrounding eosinophils (0.5 mm field diameter) (B).

Approximately a year later, the patient presented again to the ED with complaints of nausea, small-volume hematemesis, epigastric/throat burning, intermediate sharp right upper quadrant abdominal pain, and odynophagia. The patient revealed that he had not completed the full prescribed dose of acyclovir from his previous admission. Initial evaluation showed that his vital signs were as follows: heart rate (HR) of 82 (normal: 60-100 beats per minute (bpm)), blood pressure (BP) of 152/101 mmHg (normal: <120/80 mmHg), temperature of 36.5°C (normal: 36.1-37.2°C), and labs were significant for elevated white blood cells (WBC) of 21,500 cells/µL (normal: 4,500-11,000 cells/µL), hemoglobin (Hgb) of 18.2 g/dL (normal: 13.5-17.5 g/dL), creatinine of 1.7 mg/dL (normal: 0.7-1.3 mg/dL), glomerular filtration rate (GFR) of 54 mL/min/1.73 m² (normal: ≥60 mL/min/1.73 m²), and a blood urea nitrogen (BUN)/creatinine ratio of 19 mg/dL (normal: 10-20 mg/dL). CT abdomen and pelvis demonstrated thickening of the distal esophageal wall (Figure 3). Subsequent EGD revealed severe herpetiform esophagitis (Figures 4, 5A, 5B) throughout the entire esophagus with no signs of active bleeding. Biopsies and HSV immunostains confirmed the diagnosis of HE with findings of reactive changes in the form of basal cell hyperplasia (Figure 6A) and an abundance of reactive eosinophils surrounding multinucleated giant cells (Figure 6B). The patient was initiated on a course of extended-course renally dosed valacyclovir (500 mg twice a day (BID) for 14 days) in the setting of acute kidney injury, with arrangements made for close outpatient follow-up and repeat endoscopic evaluation.

## Discussion

The esophagus is the most susceptible visceral organ to HSV infection [7,9], with viral infection transmitted from direct oropharyngeal exposure and extension to the esophagus by contiguity or reactivation of HSV in the vagus nerve that then infiltrates the esophageal mucosa, affecting mostly immunocompromised individuals or immunocompetent ones who receive steroids [7,11]. HE is characterized by odynophagia, dysphagia, retrosternal chest pain, and fever. Endoscopic findings include multiple discrete, small ulcers with raised edges and central necrosis, typically in the distal esophagus. While a nucleic acid amplification test can be useful in diagnosing HE due to its high sensitivity [12,13], pathological confirmation remains the gold standard for diagnosis, with positive findings showing multinucleated giant cells [14]. In the immunocompetent population, HE is typically a self-limiting process, but a small percentage of patients, like the patient in this case, can demonstrate recurrence of HE with progression of tissue damage. For healthy patients who have not taken steroids, it is suggested that HE may be caused by tissue damage from GERD that enables HSV to infect the esophageal mucosa [9]. It is uncertain whether GERD was a contributing factor in his case due to the overlap of similar symptoms, but HE appears to occur in immunocompetent hosts at a younger age than in immunocompromised hosts [15] and is most frequently diagnosed in young males in their 30s [3], which indicates that there may be some unknown underlying biological factors that predispose certain immunocompetent patients to develop HE, as may be the case with the patient discussed in this case. Management of HSV esophagitis depends on the immune status of the patient. In immunocompetent individuals, the disease is usually self-limiting, but antiviral therapy may be considered to hasten recovery or treat patients with severe symptoms [16]. In contrast, immunocompromised patients require initiation of systemic antivirals such as acyclovir [7].

## Conclusions

This case highlights the diagnostic challenges of HE in immunocompetent individuals, where initial misdiagnosis as *Candida *esophagitis delayed appropriate antiviral therapy. Importantly, incomplete adherence to the initial acyclovir course likely contributed to inadequate viral suppression and subsequent relapse, underscoring the critical role of treatment completion and patient education. Clinicians should maintain a broad differential for esophageal ulcerations, ensure histopathologic confirmation, and emphasize close outpatient follow-up to prevent recurrence.
